# The effects of three remineralizing agents on regression of white spot lesions in children: A two-week, single-blind, randomized clinical trial

**DOI:** 10.4317/jced.53582

**Published:** 2017-05-01

**Authors:** Masoumeh Ebrahimi, Maryam Mehrabkhani, Farzaneh Ahrari, Iman Parisay, Maliheh Jahantigh

**Affiliations:** 1 Associate Professor of Pediatric Dentistry, Dental Research Center, School of Dentistry, Mashhad University of Medical Sciences, Mashhad, Iran; 2 Assistant Professor of Orthodontics, Dental Research Center, School of Dentistry, Mashhad University of Medical Sciences, Mashhad, Iran; 3 Pediatric Dentist, Private practice, Sabzevar, Iran

## Abstract

**Background:**

This study investigated the effect of three remineralizing agents on improving white spot lesions (WSLs).

**Material and Methods:**

This clinical trial included children who had at least one WSL on anterior teeth of upper or lower jaws. The participants were randomly assigned to 4 groups by treatment: 1) a cream containing casein phosphopeptide-amorphous calcium phosphate and fluoride (MI Paste Plus); 2) a cream containing hydroxyapatite and fluoride (Remin Pro); 3) a 2% sodium fluoride gel; and 4) usual home care (control). The treatment was performed for 3 times over 10 days using special trays for retaining remineralizing agents. The area and mineral content of WSLs were measured at baseline (T1) and 1 day after finishing treatment (T2). Blinding was applied for outcome assessment.

**Results:**

Eighty patients were assigned to MI Paste Plus, Remin Pro, NaF or control groups. The application of all remineralizing agents caused a significant decrease in area and a significant increase in mineral content of WSLs (*p*<0.05), whereas the control patients did not experience any significant alteration (*p*>0.05). At T2, the area of WSLs was significantly lower in three experimental groups compared to the control group (*p*=0.023), but between-group difference in mineral content of WSLs failed to achieve statistical significance (*p*=0.08).

**Conclusions:**

The in-office application of either MI Paste Plus or Remin Pro was as effective as 2% NaF for reducing area and increasing mineral content of WSLs. MI Paste Plus and Remin Pro could be recommended as suitable alternatives to NaF for managing WSLs.

** Key words:**White spot lesion, caries, casein phosphopeptide-amorphous calcium phosphate, hydroxyapatite, sodium fluoride, CPP-ACP, MPlus, Remin Pro, NaF.

## Introduction

Enamel demineralization is frequently observed in children and adolescents with poor oral hygiene. White spot lesions (WSLs) are defined as opaque enamel areas created by mineral loss from the subsurface layer of enamel. These areas are also defined as incipient or enamel caries. WSLs are the precursors for caries cavities and their milky color may cause esthetic problems that sometimes remain for several years later (1,2). Different options have been suggested for treatment of WSLs; some of them are conservative such as remineralization therapy; and others to some part aggressive such as bleaching or microabrasion (3,4).

Remineralization therapy should be considered as the best approach for treatment of WSLs. Fluoride is the most commonly-used agent for remineralization of enamel caries and its beneficial effects have been demonstrated in several investigations (5-7). Fluoride in combination with calcium and phosphate ions creates a veneer of fluoroapatite on the surface of existing enamel crystals, which not only acts as a replacement for minerals lost from the tooth structure, but also is much less soluble than the original carbonated hydroxyapatite (8). For each 2 fluoride ions, 10 calcium ions and 6 phosphate ions are required to form a unit cell of fluoroapatite [Ca10(PO4)6F2]. Therefore, in the clinical application of fluoride-containing agents, the availability of calcium and phosphate ions is the limiting factor for enamel remineralization (9). Furthermore, fluoride in higher doses may be toxic and create side effects such as fluorosis. Therefore, finding alternative remineralizing agents with minimal side effects has always been desired.

Recently, products containing calcium and phosphate ions have been developed and advocated for prevention and treatment of enamel caries. Casein phosphopeptide-amorphous calcium phosphate (CPP-ACP) is a milk product that is capable to precipitate high concentrations of calcium and phosphate ions in the vicinity of tooth structure by attaching to the tooth surface, pellicle and plaque (9,10). Therefore, the application of products containing CPP-ACP can lead to inhibition of demineralization and promotion of remineralization or most likely, a combination of both may occur (11). Recently, fluoride has been applied in association with CPP-ACP and it has been revealed that the combined application produces a synergistic effect on enamel remineralization (12-14). Tooth Mousse Plus (MI Paste Plus; GC Corporation, Tokyo, Japan) is a commercial product that contains CPP-ACP and 900 ppm fluoride (CPP-ACPF), and may provide more therapeutic effects than its precursor Tooth Mousse (MI Paste), which contains CPP-ACP alone. However, the evidence to support the benefits of CPP-ACP or CPP-ACPF on preventing early dental caries and enhancing remineralization of WSLs is still insufficient (15).

Remin Pro (VOCO GmbH, Cuxhaven, Germany) is another remineralizing paste that contains hydroxyapatite, fluoride and xylitol. It is believed that hydroxyapatite fills eroded enamel, fluoride seals dentinal tubules and xylitol acts as an antibacterial agent. The manufacturer recommended Remin Pro for controlling dentinal hypersensitivity, prevention of enamel demineralization and erosion, and promoting remineralization of carious lesions (16). However, there are few studies regarding the effectiveness of Remin Pro in regression of WSLs in children with poor oral hygiene, and its comparison with MI Paste Plus and NaF has not been performed in the clinical conditions according to the authors’ knowledge.

There are several methods to detect caries in the clinical environment; one of them is quantitative laser- or light-induced fluorescence. This technique is also capable to assess progression or regression of caries over time and thus indicating changes in enamel mineralization level (17). Fluorescent markers such as porphyrins are produced by a variety of bacterial species. Vista proof (Dürr Dental, Bietigheim-Bissingen, Germany) is a fluorescent camera developed for caries detection, and is used with DBSWIN soft-ware (Dürr Dental) for image processing. VistaCam iX (Dürr Dental) is a newer version of vista proof, which in addition to the fluorescent camera, has a head for taking intraoral images.

Different treatment protocols are available for remineralization of WSLs. In most previous studies, CPP-ACP or CPP-ACPF has been applied daily by the patients for a long period such as 1 or 3 months (18-22). A suggested in-office approach for the use of high-concentrated fluoride agents is 3 time applications over 10 days. This treatment approach may be associated with several advantages, as it eliminates the need for patient cooperation and may provide suitable results over a short period of time. However, the effectiveness of MI Paste Plus and Remin Pro when applied with this in-office procedure has not been evaluated to date. Therefore, this study was conducted to examine the effect of an in-office protocol for applying MI Paste Plus and Remin pro on mineral content and area of WSLs in 7-12 year old patients with poor oral hygiene.

## Material and Methods

This study was a parallel-group, randomized placebo-controlled clinical trial. The participants were selected from those attending the Department of Pediatric Dentistry, School of Dentistry, Mashhad University of Medical Sciences, Mashhad, Iran between April 2015 to Aguste 2015. The inclusion criteria dictated that the patients should be in the age range of 7 to 12 years, presenting at least 1 WSL on the labial surface of six permanent upper or lower anterior teeth. Patients who had systemic diseases or were sensitive to milk proteins, as well as those using drugs that cause xerostomia were excluded from the sample. The exclusion criteria also involved patients who had hypoplastic enamel and those with dentine caries on upper anterior teeth and also uncooperative patients. The study protocol was reviewed and approved by the ethics committee of Mashhad University of Medical Sciences and an informed consent document was taken from each patient’s parent/legal guardian after a brief explanation of the treatment process. The sample size for each group was calculated as n = 20, based on an alpha significance level of 0.05 and a beta of 0.2, according to the data obtained from a previous study (23).

-Interventions

The participants were randomly assigned to one of the four groups and received different treatments for WSLs. The randomization was performed by a computer-generated table of random numbers. The details of the allocated groups were recorded on cards contained in sequentially numbered, opaque, sealed envelopes. These cards were prepared by an independent person who was not involved in the study process. Once a participant was diagnosed to be in accord with the inclusion/exclusion criteria, the allocation assignment was revealed by opening the envelope by this independent person.

At the first appointment (T1), the teeth were cleaned with water slurry of pumice and rubber prophy cups. Afterwards, the patients in the study groups underwent the following treatments.

The participants in group 1 were treated by a 2% neutral sodium fluoride gel (Sultan Healthcare Inc., Englewood, New Jersey, USA). A sufficient amount of gel was inserted within a tray and the tray was then placed over the maxillary dentition and remained in place for 10 minutes. The patients were instructed to avoid eating and drinking and rinsing the mouth over 30 minutes after remineralizing therapy.

The patients in group 2 received a remineralizing cream containing CPP-ACPF (MI Paste Plus; GC Corporation, Tokyo, Japan), under the same conditions as described in group 1.

The patients in group 3 received a remineralizing cream containing fluoridated hydroxyapatite (Remin Pro, VOCO GmbH, Cux-haven, Germany), under the same conditions as described in group 1.

The participants in group 4 received no treatment and served as the control group.

The remineralizing treatments were performed 3 times over 10 days. All patients were instructed to brush twice daily using a soft-texture toothbrush and fluoridated toothpaste (Crest Cavity Protection, 1100 ppm F). The patients were also advised to avoid any supplementary fluoridated products and prevent from eating too much sugar and acidic food or drink.

-Outcomes

The patients were evaluated at the start of the study (T1) and one day after finishing the remineralizing treatment (T2). The main outcomes were any difference in the area and mineral content of WSLs.

At both T1 and T2 appointments, the teeth were first cleaned by pumice slurry and a rubber prophy cup and then a lip retractor was placed to hold the soft tissue away from the dentition. To determine the area of WSLs, the labial surfaces of the teeth with WLs were photographed by VistaCam iX (Dürr Dental, Bietigheim-Bissingen, Germany) using a special head for taking intraoral images. The photographs were taken as the patient’s head was positioned horizontally and the lens of the device was 8 centimeter away from the labial surfaces of the teeth. The intraoral images were then transported to a microstructure image processing software (MIP4 Student software; Nahamin Pardazan Asia Co, Iran). For the purpose of calibration, the mesiodistal width of the imaged tooth was imported into the software. The borders of WSLs were determined manually and their areas were calculated by the software. If a tooth had more than one WSL, the cumulative area of all WSLs was calculated and considered in the statistical analysis. Twenty sets of photographs were selected and the areas of WSLs were determined again one week later to measure intraexaminer reliability.

**Figure 1:**
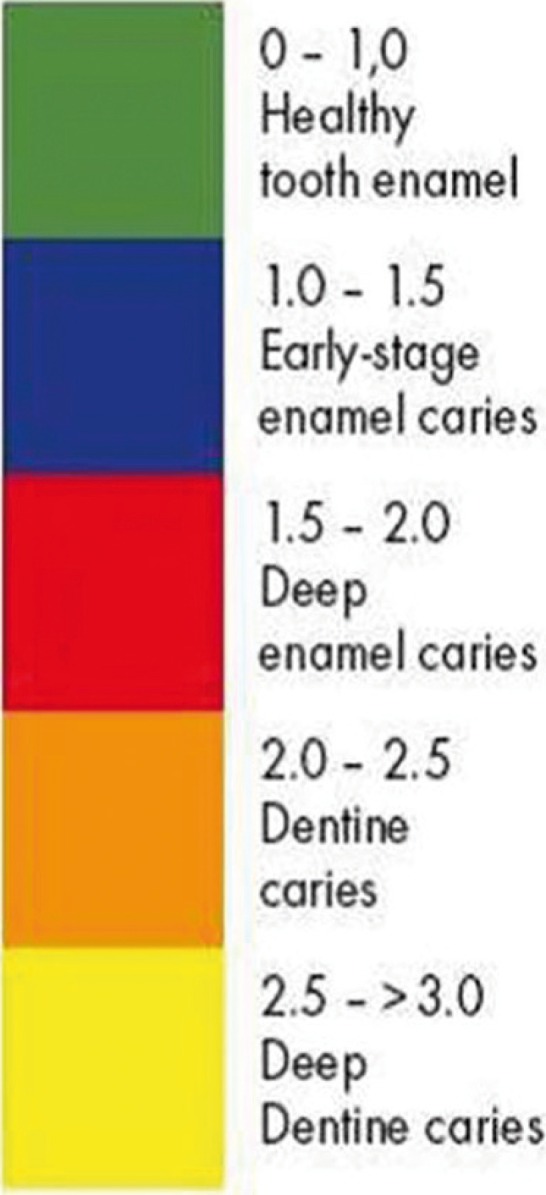
The numerical values assigned by DBSWIN software according to the stage of the caries process.

**Figure 2:**
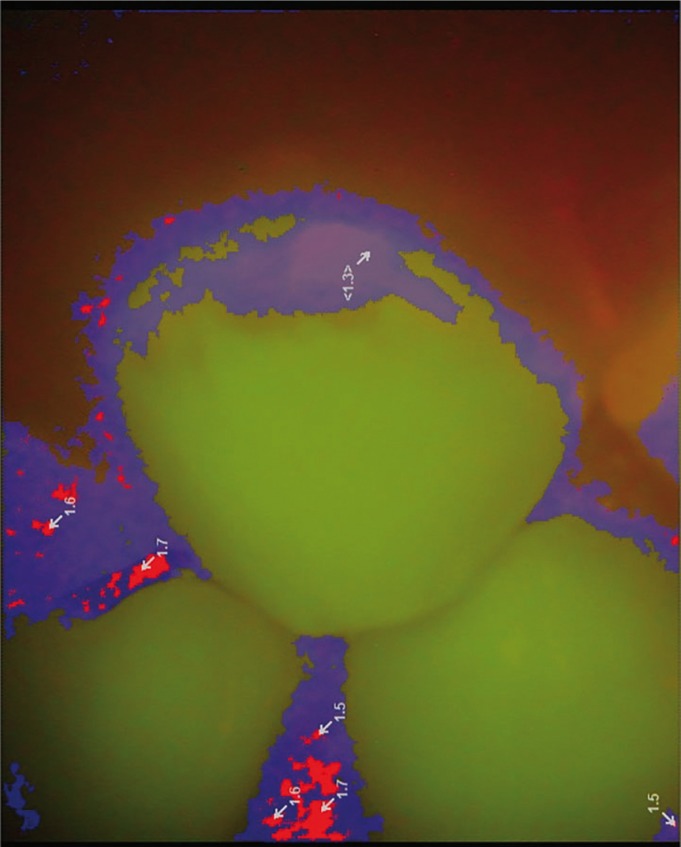
An image taken from an initial caries lesion by VistaCam iX in DBSWIN environment.

To determine the mineral content of WSLs, the labial surfaces of the selected teeth were evaluated by VistaCam iX using a special head for taking fluorescent images. The protective cover of the head was placed over the labial surface of the tooth perpendicularly and in contact with the enamel surface. The image obtained from the fluorescent reaction of the tooth was saved and processed by special software (DBSWIN Imaging Software; Durr Dental). This software creates images of 720×576 pixels. Using DBSWIN software, a numerical value from 0 to 3 is assigned to each part of the tooth proportional to the amount of mineral content on that area. Furthermore, in the fluorescent images, the teeth are illustrated in different colors from green (approximately 510 nm wavelength) to red (approximately 680 nm wavelength) according to the stage of the caries process (Fig. 1). The healthy tooth enamel is indicated in green by a value from 0 to 1.0. The value assigned to early-stage enamel caries is in the range of 1.0 to 1.5, and WSLs are illustrated in blue in the software environment. In the present study, the greatest value assigned to a tooth with WSL was recorded at each assessment interval corresponding to the lowest amount of mineral content in the tooth. Figure 2 indicates an image taken from an initial caries lesion by VistaCam iX in DBSWIN environment.

All examinations were performed by a single experienced investigator who had been trained to do these assessments before the study commencement on 10 patients, not involved in the study process. Neither the patient nor the treating clinician was blind to the group assignment. But, the investigator who assessed the outcomes was kept blinded to the group allocation.

-Statistical analysis

The Kolmogorov- Smirnov test confirmed the normal distribution of the data (*p*>0.05). A paired-sample T-Test was applied to detect significant alterations in the area and mineral content of WSLs between T1 and T2 time points in each of the study groups. One way analysis of variance (ANOVA) was run to determine any significant differences in the area and mineral content of WSLs at the start and end of treatment between the study groups. When a significant difference was noted, pairwise comparisons were made with Dunnett test. The data were analyzed by SPSS software (SPSS 16.0, Chicago, IL, USA) and the significance level was determined at *p*<0.05.

## Results

**Figure 3:**
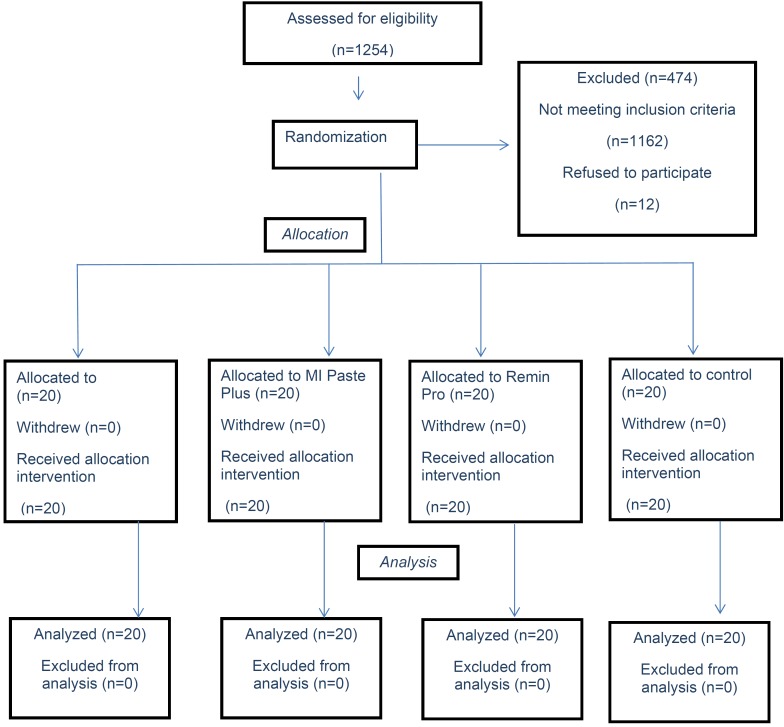
CONSORT flow diagram showing patient flow during the trial.

Eighty patients (46 girls, 34 boys; mean age, 9±2.2 years) were randomized in a 1:1:1:1 ratio to NaF, MI Paste Plus, Remin-Pro or control groups. All the participants finished the study period and had complete records for statistical analysis (Fig. 3). No significant difference was observed in age or sex of the participants among the study groups (*p*>0.05).

Using the 20 sets of repeated measurements, the correlation coefficient for detecting the area of WSLs was 0.95, indicating excellent intra-examiner reliability.

 Table 1 presents the descriptive statistics (mean, standard deviation) regarding the area of WSLs in the study groups at baseline (T1) and one day after the end of the remineralization treatment (T2). Comparison of surface area of WSLs between T1 and T2 time points in each group revealed a significant reduction in the area of WSLs in patients underwent treatment with NaF, MI Paste Plus and Remin Pro (*p*<0.05), whereas in the control group no significant improvement in the area of WSLs occurred between the two assessments (*p*>0.05) ( Table 1). Between-group comparison by ANOVA revealed no significant difference in the area of WSLs at T1 (*p*=0.353), whereas at T2 time point, the difference between groups was significant (*p*=0.023). Pairwise comparison by Dunnett test exhibited that the area of WSLs was comparable in NaF, Remin Pro, and MI Paste Plus groups (*p*>0.05), and all showed significantly lower demineralized area compared to the control group (*p*=0.009, *p*=0.005 and *p*=0.003, respectively).

**Table 1:**
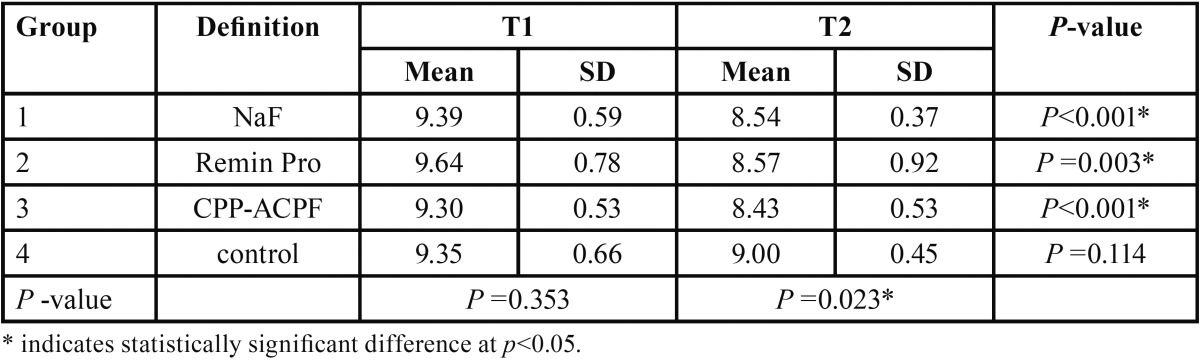
The mean, standard deviation (SD) and the results of the statistical analysis regarding the area of WSLs (mm2) in the study groups at the start (T1) and end (T2) of the experiment.

 Table 2 indicates the summary statistics (mean, standard deviation) for mineral content of WSLs in the study groups at both assessment intervals. When the alteration in mineral content of WSLs was compared between T1 and T2 time points in each group, it was revealed that the mineral content of WSLs increased significantly in patients underwent treatment with NaF, MI paste Plus and Remin Pro (*p*<0.05), whereas the increase in mineral content of WSLs in the control group was not significant (*p*>0.05) ( Table 2). Between-group comparison by ANOVA revealed no significant difference in mineral content of WSLs either at T1 (*p*=0.143) or at T2 time points (*p*=0.08) among the study groups.

**Table 2:**
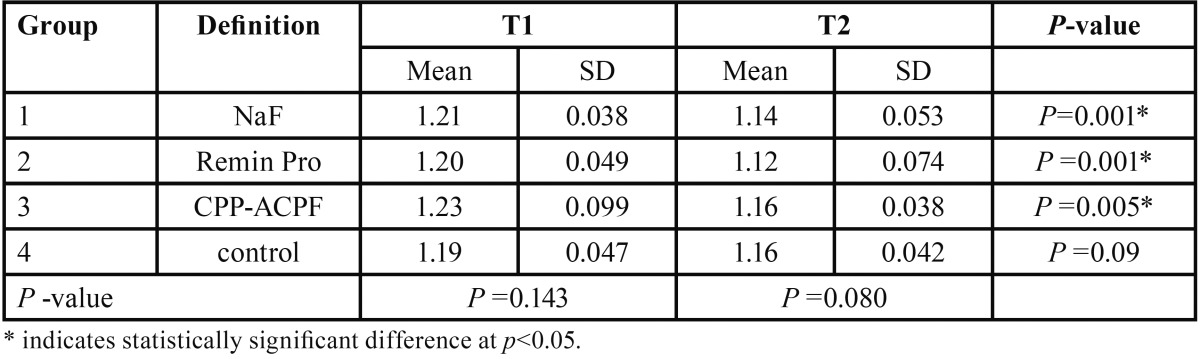
The mean, standard deviation and the results of the statistical analysis regarding the mineral content of WSLs in the study groups at the start (T1) and end (T2) of the experiment.

No harm was observed over the period of the experiment.

## Discussion

The present study investigated the effectiveness of MI Paste Plus, Remin Pro and NaF for improving area and mineral content of WSLs in children. The treatment regimen applied in this study was 3 times application of the remineralizing agent over 10 days. The 10-day treatment period was chosen in this study in order to assess a fast approach for managing WSLs. We preferred the in-office procedure, because children in the age range of this study may be uncooperative in effective brushing and use of remineralizing agents. Furthermore, the use of special trays over dentition ensured an even and long contact of the remineralizing material with the tooth structure.

In the present study, VistaCam iX was employed for assessing mineral content of WSLs and taking intraoral photographs. This device is small and portable and thus easily applied in the clinical situation. It has been demonstrated that fluorescent-based systems are suitable for detection of caries lesions and assessing alteration in mineral level of tooth structure (17,24-26). Jablons-ki-Momeni *et al.* (27) exhibited that VistaCam iX provides high reproducibility and good performance for caries detection at various stages of the disease process. Al-Khateeb *et al.* (25) found a significant correlation between fluorescent changes and mineral loss, and thus recommended the fluorescent camera as a sensitive device for assessing the severity of incipient enamel lesions. In order to minimize measurement errors, the intraoral photographs were taken in similar lighting conditions and the camera was positioned at the same angular and linear position to the tooth surface. The use of MIP4 Student software provided precise measurement for area of WSLs in photographs (28,29).

In the present study, all the experimental groups experienced significant decrease in area of WSLs over the course of the experiment, whereas the control group did not show any significant improvement. Furthermore, the area of WSLs was significantly lower in the three experimental groups compared to the control group at the end of the study (T2). These results were interesting as the use of remineralizing agents lead to the regression of WSLs even in the short treatment period of this study. The reduction in the area of WSLs is not only important from the point of limiting caries lesion, but also from the esthetic point of view, especially in the anterior part of the dentition, where WSLs produce esthetic problems. In the control group, a small and insignificant improvement in the area of WSLs was observed over time, possibly due to the natural remineralization phenomenon, which results in partial improvement of WSLs without any treatment.

Another variable that was measured in the present study was the mineral content of WSLs, as represented by VistaCam iX apparatus. The values represented by this device are correlated with the mineralization level of tooth structure. The normal enamel is usually displayed by 1, whereas WSLs indicate values in the range of 1-1.5 in the software environment. Therefore, any reduction in the values represented by VistaCam iX is correlated with the increase in mineral content of tooth structure. In the present study, a significant increase in mineral content of WSLs occurred after remineralizinng therapy in three experimental groups, implying the effectiveness of CPP-ACP + fluoride and hydroxyapatite + fluoride as well as 2% NaF in restoring the minerals lost from the tooth structure. The increase in mineral content of WSLs in the control group was small and not statistically significant. This small improvement in mineral content of the control group should be again attributed to the natural remineralization of WSLs in the presence of saliva and oral hygiene instructions. Although the experimental groups experienced significant increase in mineral content of WSLs, the difference between groups failed to achieve statistical significance at the end of the study period (*p*=0.08). This may be related to the low frequency of applying remineralizing agents over the short period of this study (3 times over 10 days). Another possibility is that the apparatus employed in this study (VistaCam iX) was not enough precise to detect small dif-ferences in mineral content of WSLs among the study groups.

In the present study, both MI Paste Plus (containing CPP-ACP and fluoride) and Remin Pro (containing hydroxyapatite and fluoride) were as effective as 2% NaF for reducing the area and increasing the mineral content of WSLs over time, and thus they could be recommended as suitable alternatives for high concentrated fluoride agents. In contrast, Salehzadeh Esfahani *et al.* (30) exhibited that CPP-ACP had significantly better outcomes compared to Remin Pro and sodium fluoride varnish in increasing microhardness of artificially-induced enamel lesions. It is believed that MI Paste Plus can maintain a state of supersaturation of calcium and phosphate over the enamel surface, and the fluoride content in this product has a synergistic effect with CPP-ACP, increasing its remineralizing potential (12,14). Remin Pro is a remineralizing water-based agent that can increase the insertion of hydroxyapatite and fluoride into enamel lesions and thus enhancing remineralization. In this study, the application of NaF gel also lead to remineralization, possibly due to the formation of alkali compounds containing fluoride around enamel surface, which prevent demineralization and enhance remineralization of dental structure. The findings of this study indicate that in patients with poor oral hygiene, any natural improvement in area and mineral content of WSLs is negligible, and remineralization treatment should be considered as a viable option for incipient caries lesions. Either MI Paste Plus or Remin Pro could be considered as suitable alternatives for 2% NaF in treatment of WSLs. The use of these alternatives may have some advantages over NaF as they are good-tasting and are not toxic in higher dosage, and thus they could be applied safely by either patients or clinicians. Although the treatment period in this study was only 10 days, but the results were promising and thus this protocol can be recommended in the clinical conditions for patients with extensive enamel caries who may benefit from a rapid remineralization program.

The outcomes of this study are in agreement with the results of several previous investigators who found significant regression of WSLs after the use of products containing CPP-ACP or CPP-ACPF (18,19). Robertson *et al.* (19) indicated that the application of MI Paste Plus prevented the development of WSLs during orthodontic treatment and reduced the number of WSLs already pre-sent, whereas the placebo paste had no significant effect on prevention and treatment of enamel caries lesions. Baily *et al.* (18) showed that significantly more WSLs regressed after 12-week application of a remineralizing cream containing CPP-ACP compared with a placebo. In these studies (19,20), the period of treatment with CPP-ACP or CPP-ACPF was 1 to 3 months, but the present study provided satisfactory results over a period of just 10 days. In contrast to the findings of this study, some clinical trials (20-22) reported that the application of a cream containing CPP-ACP or CPP-ACPF was not superior to brushing with a fluoride tooth paste for regression of WSLs. The difference between the results of this study and those of previous studies may be related to the use of fluoride-free CPP-ACP by some authors (21). Another factor that could affect the results is the volume of the remineralizing agent and its close contact with WSLs. Several studies recommended a pea-sized amount or 1 g of CPP-ACP ap-plied by a finger or brush (21,22), but in this study, special trays were placed over sufficient amounts of remineralizing agents, providing close proximity to enamel structure. Furthermore, the duration of applying remineralizing agent was 10 minutes, which was longer than that used in most previous investigations (21,22), and the treatment protocol was not dependent on patient cooperation, as it was accomplished in the dental office.

The limitation of this study was its short duration and the lack of blinding for the patients and the treating clinician, because in-dustrial products were used. However, the outcome assessor was blind, so the risk of biases was minimized. Further studies are warranted to assess the effect of long term use of remineralizing agents on prevention and regression of WSLs in children. The use of supplementary instruments to measure the degree of improvement of incipient caries lesions is also helpful. Although the re-mineralizing effects of MI Paste Plus, Remin Pro and NaF were similar in the present study, but further studies with larger sample size and longer period of treatment are required to ensure the lack of significant difference in performance of these remineralizing agents.

## Conclusions

Under the conditions used in this clinical trial.

1- The application of MI Paste Plus, Remin Pro or NaF for three times over 10 days was effective in reducing the area and increasing the mineral content of WSLs in 7-12 years old children, whereas the control group did not show any significant improvement. Therefore, remineralization therapy should be recommended in children presenting incipient caries lesions.

2- There was no significant difference between MI Paste Plus, Remin Pro and NaF regarding their effectiveness in regression of WSLs in children. Considering the lower side effects of MI Paste Plus and Remin Pro, these products could be recommended as suitable alternatives for high concentrated fluoride agents.
